# Exploring the Prominent and Concealed Inhibitory Features for Cytoplasmic Isoforms of Hsp90 Using QSAR Analysis

**DOI:** 10.3390/ph15030303

**Published:** 2022-03-01

**Authors:** Magdi E. A. Zaki, Sami A. Al-Hussain, Syed Nasir Abbas Bukhari, Vijay H. Masand, Mithilesh M. Rathore, Sumer D. Thakur, Vaishali M. Patil

**Affiliations:** 1 Department of Chemistry, Faculty of Science, Imam Mohammad Ibn Saud Islamic University, Riyadh 13318, Saudi Arabia; sahussain@imamu.edu.sa; 2 Department of Pharmaceutical Chemistry, College of Pharmacy, Jouf University, Al Jouf 72388, Saudi Arabia; sbukhari@ju.edu.sa; 3 Department of Chemistry, Vidya Bharati Mahavidyalaya, Amravati 444 602, Maharashtra, India; mithsrathore@gmail.com; 4 Department of Chemistry, RDIK and NKD College, Badnera-Amravati 444 701, Maharashtra, India; sdthakur11@gmail.com; 5 Department of Pharmaceutical Chemistry, KIET School of Pharmacy, KIET Group of Institutions, Delhi-NCR, Ghaziabad 201206, Uttar Pradesh, India; vaishuwise@gmail.com

**Keywords:** Hsp90, cancer, QSAR, machine learning, pharmacophores

## Abstract

Cancer is a major life-threatening disease with a high mortality rate in many countries. Even though different therapies and options are available, patients generally prefer chemotherapy. However, serious side effects of anti-cancer drugs compel us to search for a safer drug. To achieve this target, Hsp90 (heat shock protein 90), which is responsible for stabilization of many oncoproteins in cancer cells, is a promising target for developing an anti-cancer drug. The QSAR (Quantitative Structure–Activity Relationship) could be useful to identify crucial pharmacophoric features to develop a Hsp90 inhibitor. Therefore, in the present work, a larger dataset encompassing 1141 diverse compounds was used to develop a multi-linear QSAR model with a balance of acceptable predictive ability (Predictive QSAR) and mechanistic interpretation (Mechanistic QSAR). The new developed six-parameter model satisfies the recommended values for a good number of validation parameters such as R2tr = 0.78, Q2LMO = 0.77, R2ex = 0.78, and CCCex = 0.88. The present analysis reveals that the Hsp90 inhibitory activity is correlated with different types of nitrogen atoms and other hidden structural features such as the presence of hydrophobic ring/aromatic carbon atoms within a specific distance from the center of mass of the molecule, etc. Thus, the model successfully identified a variety of reported as well as novel pharmacophoric features. The results of QSAR analysis are further vindicated by reported crystal structures of compounds with Hsp90.

## 1. Introduction

Cancer kills; therefore, medicinal chemists are continuously trying to develop therapeutic agents that could retard the growth of cancer cells. In cancer cells, a protein Hsp90 (heat shock protein 90, also known as HSPC) is overexpressed [1]. It is a highly conserved, non-fibrous, and chaperone protein with a key role in many cellular processes like proper folding of other proteins, apoptosis, cell cycle control, cell viability, and degradation and signaling events [1,2,3,4,5,6]. As the name indicates, Hsp (heat shock proteins) shield cells when stressed by higher temperatures. The number “90” comes from the fact that it weighs about 90 kDa. There are two isoforms of Hsp90: Hsp90α (the inducible form) and Hsp90β (the constitutive form), which are found in cytoplasm and share 85% sequence identity [1,2,3,4,5,6]. These two isoforms are like flexible biological catalysts and interact with a good number of newly synthesized proteins, such as Akt2, CDKs, PKC, MAP kinases, steroid receptors, BCL-6, CAR, p53, Oct4, etc., to avoid their aggregation or mistakes in their folding [6]. Despite a crucial role, in cancer cells, these are responsible for the stabilization of a number of oncoproteins required for tumor growth, leading to their overexpression [1,2,3,4,5,6]. Consequently, Hsp90 is an attractive target for developing a drug for cancer.

**Figure 1:**
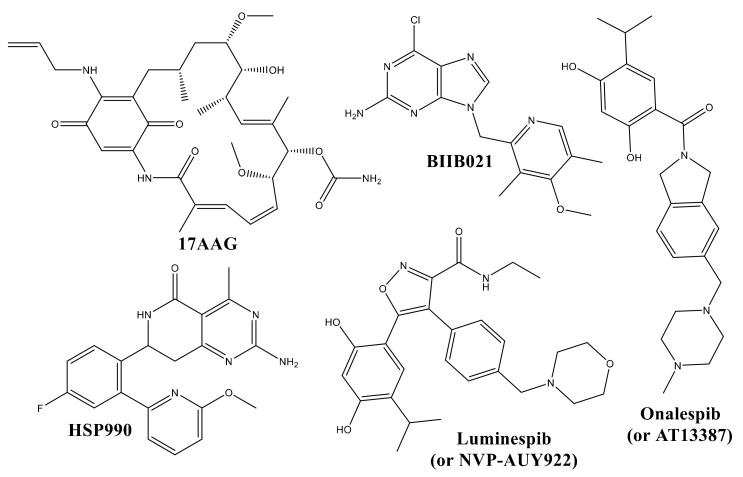
Different clinical trial candidates as inhibitors of Hsp90.

The majority of Hsp90 inhibitors occupy the ATP (adenosine tri-phosphate) pocket in the N-terminal domain of Hsp90, leading to limited ATPase activity [1,2,3,4,5,6]. At present, several natural and semi-synthetic Hsp90 inhibitors (see Figure 1) are in different stages of clinical trials for a variety of cancers [2,3,7,8,9].

Unfortunately, several inhibitors have shown hepatotoxicity and ocular toxicity [2,10]; consequently, there is a need to modify them with retention of activity against Hsp90, which could be achieved on knowing the structural features responsible for their Hsp90 inhibitory activity. A simple, cost-effective, and faster yet effective strategy to know crucial pharmacophoric features is to use QSAR (Quantitative Structure–Activity Relationships), a successful, contemporary, and widely used branch of computer assisted-drug designing [11,12,13,14,15,16]. 

In QSAR analysis, generally, a good number of inhibitors are analyzed using a suitable technique like machine learning, deep learning, etc. There are two main advantages of using the QSAR approach [11,17,18]: (a) the analysis helps to identify the prominent structural features or patterns that influence the bioactivity profile of molecules (Mechanistic interpretation or Qualitative QSAR), and (b) the analysis could be used to predict the desired bioactivity of a molecule prior to its synthesis and lab testing (Predictive ability or Predictive QSAR). Therefore, many researchers prefer QSAR as a method of choice for drug/lead optimization. Nowadays, a QSAR analysis with a balance of mechanistic interpretation with predictive ability is highly preferred. 

The literature survey reveals that QSAR analyses have been reported for Hsp90, but they are either based on a small dataset, lack general applicability, have poor predictive ability, are deficient of a mechanistic interpretation, or a combination of these factors, which limit their use [9,19,20,21,22]. Therefore, in the present work, we accomplished QSAR analysis for a larger and diverse dataset of Hsp90 inhibitors, and followed the OECD (Organization for Economic Cooperation and Development) guidelines while developing a QSAR model to have a balance of mechanistic interpretation with predictive ability.

## 2. Results

The exhaustive and heuristic search resulted in the development of a six-descriptor-based QSAR model (see model-A), which was subjected to thorough statistical validation for internal and external validations.

**Model-A:** pIC_50_ (M) = 3.903 (± 0.134) + 0.101 (± 0.013) × **com_ringChyd_4A** + 0.433 (± 0.058) × **faroCN2B** + 0.714 (± 0.214) × **aroCminus_sumpc** + 0.065 (± 0.005) × **aroC_aroN_5B** + 0.266 (± 0.048) × **fringNsp3C5B** + 0.59 (± 0.082) × **da_amdN_6B**

Statistical validation of model-A: 

N_tr_ = 915, N_ext_ = 226, R^2^_tr_ = 0.779, R^2^_adj._ = 0.777, R^2^_tr_ − R^2^_adj._ = 0.002, LOF = 0.244, Kxx = 0.219, ΔK = 0.122, RMSE_tr_ = 0.487, MAE_tr_ = 0.404, RSS_tr_ = 217.321, CCC_tr_ = 0.876, s = 0.489, F = 533.134, R^2^_cv_ (Q^2^loo) = 0.775, R^2^-R^2^_cv_ = 0.004, RMSE_cv_ = 0.491, MAE_cv_ = 0.407, PRESS_cv_ = 220.839, CCC_cv_ = 0.874, Q^2^_LMO_ = 0.775, R^2^_Yscr_ = 0.007, Q^2^_Yscr_ = −0.009, RMSE_ex_ = 0.474, MAE_ex_ = 0.383, PRESS_ext_ = 50.675, R^2^_ex_ = 0.779, Q^2^-F^1^ = 0.778, Q^2^-F^2^ = 0.778, Q^2^-F^3^ = 0.791, CCC_ex_ = 0.876, R^2^-ExPy = 0.779, R’_o_^2^ = 0.727, k’ = 0.989, 1−(R^2^/R’_o_^2^) = 0.066, r’^2^m = 0.602, R_o_^2^ = 0.779, k = 1.005, 1 − (R^2^ − ExPy/R_o_^2^) = 0, r^2^m = 0.766 

**Figure 2:**
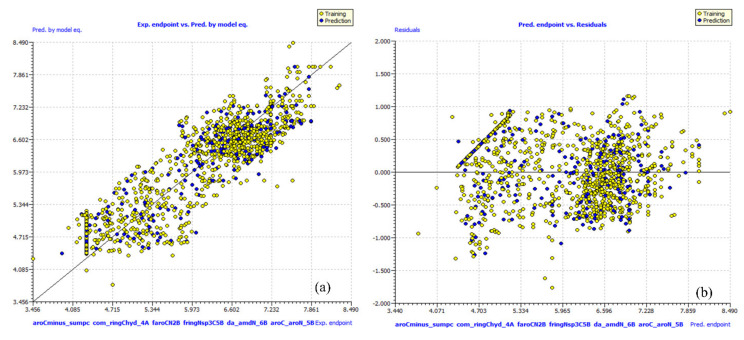

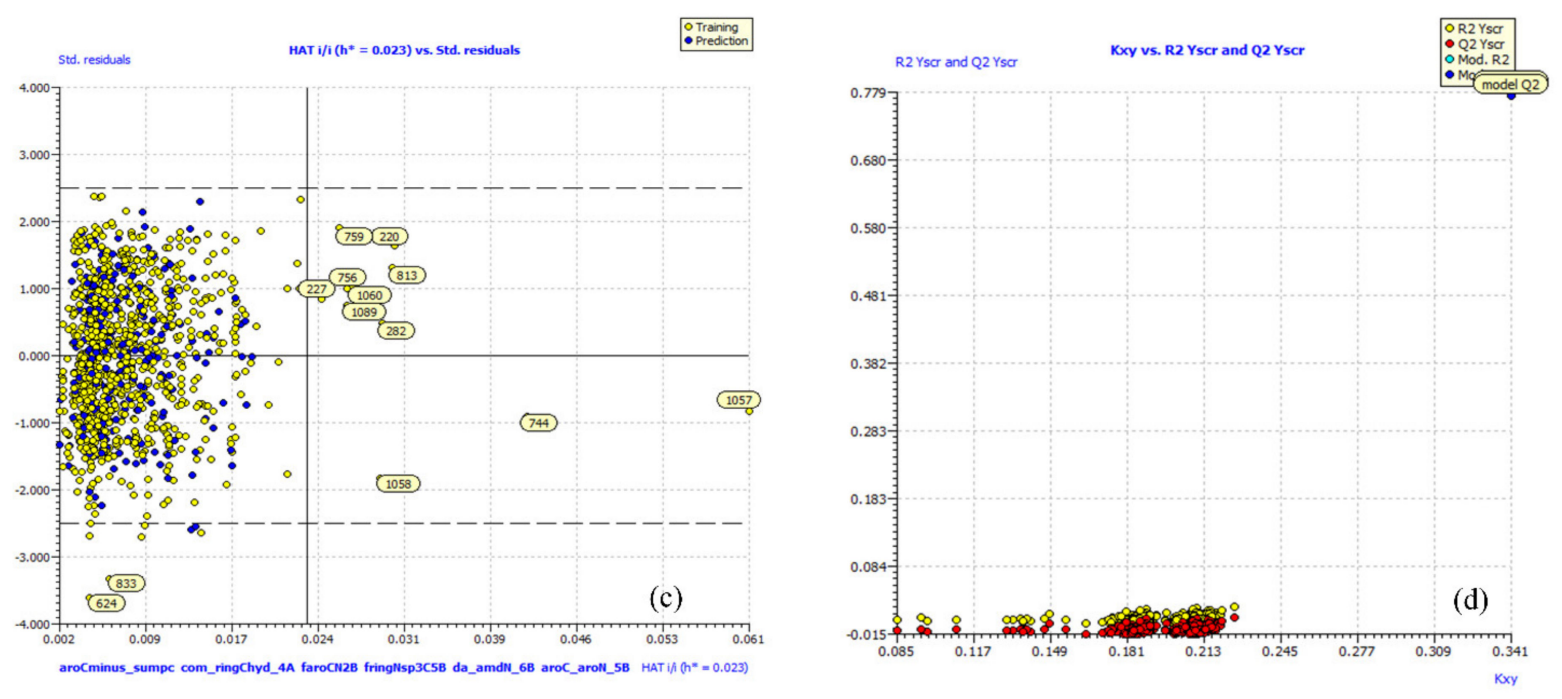
Different graphs associated with model-A: (**a**) experimental vs. predicted pIC_50_ (the solid line represents the regression line), (**b**) experimental vs. residuals, (**c**) Williams plot for applicability domain (the vertical solid line represents h* = 0.023 and horizontal dashed lines represent the upper and lower boundaries for applicability domain), and (**d**) Y-randomization.

Different researchers have recommended the above statistical parameters to judge the robustness and external predictive ability of a QSAR model [11,12,13,14,15,16,23,24,25,26,27,28,29,30,31]. The formula to calculate them is available in the Supplementary Materials. It is clear that model-A fulfils the recommended threshold for many validation parameters and other criteria. A high value of different parameters like R^2^_tr_ (coefficient of determination), R^2^_adj._ (adjusted coefficient of determination), and R^2^_cv_ (Q^2^loo, cross-validated coefficient of determination for leave-one-out), R^2^_ex_ (external coefficient of determination), Q^2^−F^n^**,** and CCC_ex_ (Concordance Correlation Coefficient), etc., and a low value of LOF (lack-of-fit), RMSE_tr_ (root mean square error), MAE_tr_ (mean absolute error), R^2^_Yscr_ (R^2^ for Y-scrambling), etc. along with the different graphs (see Figure 2) associated with the model indicate that the model possesses statistical robustness with excellent internal and external predictive ability as well as free from chance correlations. Additionally, the Williams plot specifies that the model is statistically acceptable (see Figure 2d). Therefore, it fulfils all the OECD recommended guidelines for creating a useful QSAR model.

## 3. Discussion

### Mechanistic Interpretation of QSAR Model

A very crucial aspect of a useful QSAR analysis is to gain deep insight into the pharmacophore or structure-oriented linking of molecular descriptors [17,32]. This not only helps throughout the drug discovery process, but also expands the information and understanding of mechanistic aspects of different types of molecules. Though, in the present work, a specific molecular descriptor was used to equate the pIC_50_ values of different molecules, but an extending or reverse influence of unknown factors or other molecular descriptors, having a dominant effect in deciding the final pIC_50_ value of a molecule, cannot be ignored. To simplify, a single molecular descriptor (in turn structure feature) cannot decide the overall experimental pIC_50_ value of a molecule. In other words, the effective use of an appropriately validated QSAR model depends on the synchronous consideration of all constituent molecular descriptors. Interestingly, in model-A, all the molecular descriptors have positive coefficients, which indicates that increasing their value could result in a better Hsp90 inhibitory activity. 

The descriptor **com_ringChyd_4A** represents the total number of hydrophobic ring carbons, having partial charge in the range ±0.2, within 4Å from the com (center of mass) of the molecule. From this, it appears that mere total number of ring carbons is very important, but replacing **com_ringChyd_4A** with **nringC** (number of ring carbon atoms) or **naroC** (number of aromatic carbon atoms) significantly reduced the statistical performance of the model (R^2^ = 0.72). To add further, **com_ringChyd_4A** has a positive correlation of R = 0.488 with pIC_50_, whereas **nringC** and **naroC** have a correlation of R = 0.461 and 0.405, respectively. **com_ringChyd_3A** and **com_ringChyd_5A** represent the total number of ring carbons, having partial charge in the range ±0.2, within 3Å and 5Å from the com (center of mass) of the molecule, respectively. Replacement of **com_ringChyd_4A** with **com_ringChyd_3A** or **com_ringChyd_5A** resulted in slightly reduced performance of the model with R^2^ = 0.75 and 0.76, respectively. This indicates that the optimum distance is 4Å.

**Figure 3:**
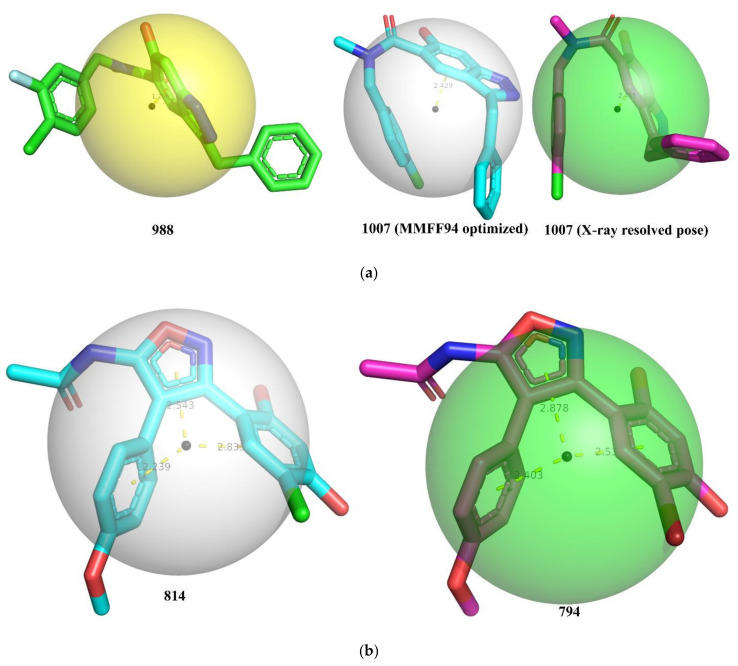
Depiction of **com_ringChyd_4A** using different molecules: (**a**) molecules 988, 1007 (MMFF94 optimized), and 1007 (X-ray resolved dock pose from pdb 6EY8); (**b**) molecules 794 and 814 (both X-ray-resolved poses from pdb 5XR9 and 4LWE, respectively). The small black sphere represents the com (center of mass) and the bigger transparent sphere represents the distance of 4Å from the center of mass. The dotted yellow line represents the distance (Å) of com from the centers of the different nearest rings.

The importance of hydrophobic ring carbon atoms is supported by the X-ray-resolved structure of a good number of Hsp90 inhibitors because the active site of Hsp90 consists of lipophilic side chains of Leu48, Ile91, Val186, Leu315, Ile388, and Val391 [33,34], which favors the presence of hydrophobic moiety in the inhibitors. For example, a comparison of molecule 988 (pIC_50_ = 6.009, **com_ringChyd_4A** = 10) with 1007 (pIC_50_ = 6.481, **com_ringChyd_4A** = 15) highlights the importance of **com_ringChyd_4A**. Another pair of molecules, viz. 794 and 814, also supports this observation. The molecular descriptor **com_ringChyd_4A** is depicted in Figure 3 for different molecules. 

From Figure 3, it is clear that the lowest energy conformer of molecule 988 has **com_ringChyd_4A** = 10 due to the closer presence of com (distance 1.206 Å) to the benzene ring of indazole ring. In case of molecule 1007 (MMFF94-optimized and X-ray-resolved pose from pdb 6EY8), the com is located slightly away from the benzene ring of Indazole ring at a distance > 2.40 due to specific conformation, thereby increasing the value of **com_ringChyd_4A** to 15. This could be a plausible reason for the difference in the bioactivity of these two compounds. Similarly, a better Hsp90 inhibitory activity of molecule 794 than 814 could be attributed to difference in their **com_ringChyd_4A** values.

Another molecular descriptor that has a positive effect on Hsp90 activity is **faroCN2B**, which signifies the presence of nitrogen exactly at two bonds from aromatic carbon atoms. If the same nitrogen atom is also present at two or less bonds from any other aromatic carbon atom, then it was excluded while calculating **faroCN2B**. This descriptor highlights the importance of nitrogen atoms separated from aromatic ring (Benzene, etc.) by two bonds. As the majority of nitrogen atoms act as either an H-bond donor or acceptor; therefore, the presence of nitrogen atoms in the vicinity of aromatic rings could be useful in enhancing interactions with the polar residues of receptor (Hsp90). Additionally, the descriptor further points out the crucial role played by the aromatic rings undoubtedly due to their lipophilic nature. Taken together, the descriptor **faroCN2B** signifies the importance of two important structural features: aromatic rings and their vicinal nitrogen atoms.

**Figure 4:**
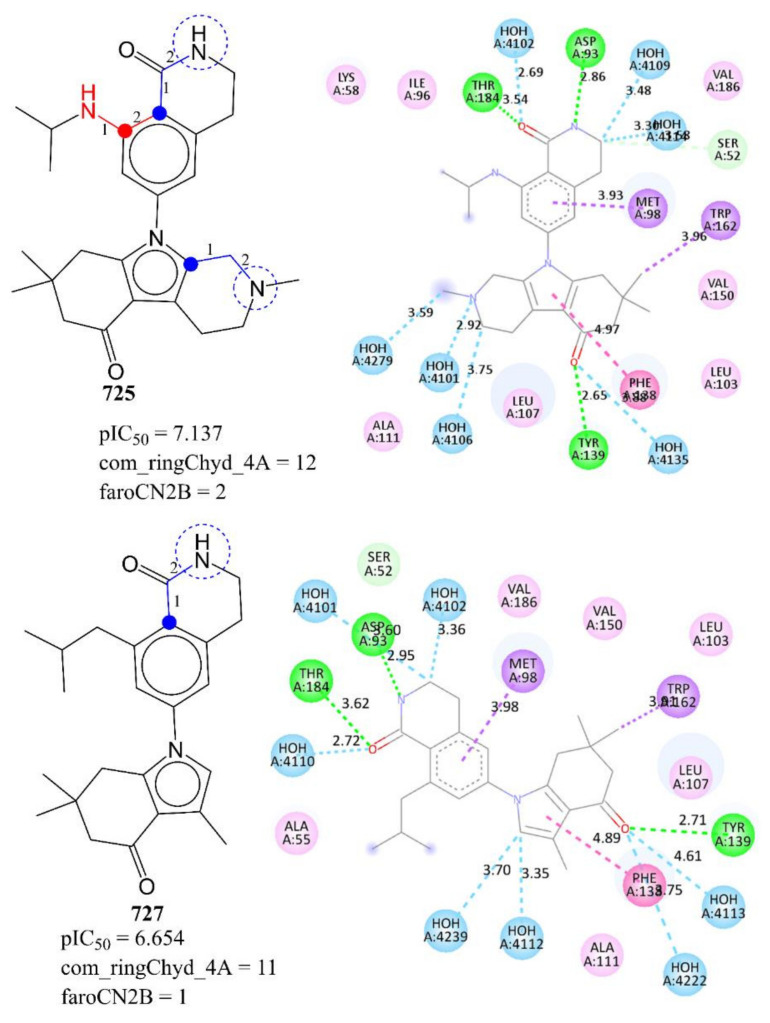
Depiction of **faroCN2B** using representative examples only.

This observation is confirmed when we compare the X-ray-resolved structures of molecule 727 (pIC_50_ = 6.654, **faroCN2B** = 1, pdb = 4O09) with 725 (pIC_50_ = 7.137, **faroCN2B** = 2, pdb = 4O05) depicted in Figure 4. The nitrogen atoms responsible for **faroCN2B** are highlighted by blue dotted circles. From Figure 4, it is clear that the aromatic ring B of both the molecules is responsible for hydrophobic interactions with the residue Met98. The nitrogen atom of ring A present in both the molecules is not only a constituent of **faroCN2B,** but also responsible for H-bonding with the residue Asp93. Thus, such a combination of aromatic carbons and nitrogen is highly beneficial to enhance the interactions with the receptor. In case of molecule 733, an additional nitrogen atom is present in ring F, which is a constituent of **faroCN2B**, and responsible for the H-bond interaction with the nearby water molecule. Thus, the present QSAR analysis revealed an important structural feature, which is also visible in X-ray-resolved structures of the same inhibitors with the same target enzyme Hsp90.

A comparison of the following pairs of molecules further vindicates the importance of **faroCN2B** in determining the bioactivity: 213 (pIC_50_ = 6.523, **faroCN2B** = 2) with 212 (pIC_50_ = 6.469, **faroCN2B** = 1) and 758 (pIC_50_ = 7.444, **faroCN2B** = 2) with 759 (pIC_50_ = 7.569, **faroCN2B** = 3). 

The importance of aromatic carbon atoms is further emphasized with the presence of **aroCminus_sumpc** as a constituent variable of model-A. The molecular descriptor **aroCminus_sumpc** represents the sum of partial charges on negatively charged aromatic carbon atoms. The positive coefficient for **aroCminus_sumpc** indicates that the higher the value of this descriptor, the better the activity profile. The sum of partial charges on negatively charged aromatic carbon atoms will always be negative; therefore, in reality, this descriptor actually decreases the pIC_50_ value. Further, the replacement of **aroCminus_sumpc** by **aroCplus_sumpc** (sum of partial charges on positively charged aromatic carbon atoms) led to a model with almost identical statistical performance (R^2^_tr_ = 0.772, Q^2^_LMO_ = 0.767, R^2^_ex_ = 0.78, CCC_ex_ = 0.876). In fact, **aroCplus_sumpc** has a better correlation (R = 0.33) with pIC_50_ than **aroCminus_sumpc** (R = 0.10). From this it is clear that, if aromatic carbons are positively charged than the molecule possesses better Hsp90 inhibitory activity. Therefore, the best strategy is to attach atoms or groups that enhance lipophilic and mild polar interactions with the receptor (for example -Cl, etc.) to the aromatic carbon atoms. In short, substituted aromatic rings are preferable for better activity. This observation is supported by comparing following pairs of molecules: 2 with 3, 1054 with 1059, and 214 with 212. 

**aroC_aroN_5B**, which represents the total number of aromatic carbon atoms within five bonds from aromatic nitrogen atoms, again points out the key role played by aromatic carbon atoms in deciding Hsp90 inhibitory activity. It also underlines the usefulness of aromatic nitrogen atoms. This descriptor has a positive correlation with pIC_50_ with R = 0.63. Therefore, an increase in number of aromatic carbon atoms within five bonds from aromatic nitrogen atoms leads to better Hsp90 inhibitory activity. The following pairs of the molecules support this observation: 888 (pIC_50_ = 7.523, **aroC_aroN_5B** = 22) with 887 (pIC_50_ = 6.046, **aroC_aroN_5B** = 20) and 107 (pIC_50_ = 5.953, **aroC_aroN_5B** = 13) with 108 (pIC_50_ = 4.874, **aroC_aroN_5B** = 10), to mention a few. Further, the 50 most active molecules possess relatively higher value of **aroC_aroN_5B** (range 8–17) than the 50 least active molecules (range 0–8). 

**fringNsp3C5B** stands for the number of sp^3^-hybridized carbon atoms exactly at five bonds from the ring nitrogen atom. If the same sp^3^-hybridized carbon atom is also present at four or less bonds from any other ring nitrogen atom, then it was excluded while calculating **fringNsp3C5B**. It is interesting to note that the 50 most active molecules, except molecule 618, possess at least one or more of such a combination of carbon and ring nitrogen, whereas the 50 least active molecules either lack it or have **fringNsp3C5B** = 1. In the majority of compounds, the sp^3^-hybridized carbon atoms are present either as a linker between two rings or as a substituent, which therefore enhances conformational flexibility of the molecule to adopt a bioactive conformer or lipophilic characters of the molecule. A comparison of 895 (pIC_50_ = 7.071, **fringNsp3C5B** = 2) with 896 (pIC_50_ = 6.777, **fringNsp3C5B** = 1), 859 (pIC_50_ = 7.237, **fringNsp3C5B** = 2) with 896 (pIC_50_ = 7.071, **fringNsp3C5B** = 1), 326 (pIC_50_ = 6.921, **fringNsp3C5B** = 1) with 328 (pIC_50_ = 7.046, **fringNsp3C5B** = 2), and 412 (pIC_50_ = 7.155, **fringNsp3C5B** = 1) with 411 (pIC_50_ = 6.959, **fringNsp3C5B** = 0) and 410 (pIC_50_ = 6.854, **fringNsp3C5B** = 0) confirms the importance of **fringNsp3C5B** in deciding the activity.

**Figure 5:**
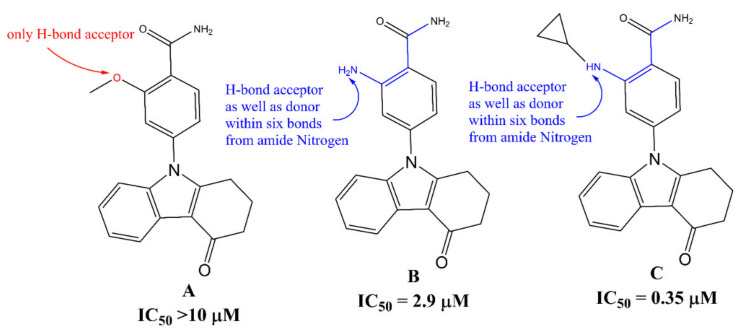
Pictorial representation of **da_amdN_6B** using representative examples only.

A molecular descriptor that identifies the relation of total number amide nitrogen atoms within six bonds from the H-bond donor and acceptor atoms is **da_amdN_6B**. In the majority of compounds in the present dataset, the amide group is present as a substituent on aromatic ring or as a linker between two rings. The descriptor **da_amdN_6B** suggests the significance of amide group and its correlation with the H-bond donor and acceptor atoms. This observation is confirmed on comparing molecule **A** with molecules **B** and **C** (see Figure 5).

A good number of researchers have also pointed out that the amide group is crucial for Hsp90 inhibitors to establish H-bonding with residues of the active site (see pdb 4AWO). For example, Zhao et al. [4] pointed out that the distance between the nitrogen atoms on the piperidine ring and the amide are important for Hsp90 inhibition. Similarly, Baruchello and co-workers [35] studied a library of 3,4-isoxazole diamides for Hsp90 binding and found that a substantial reduction in Hsp90 binding affinity when the amide was replaced with substituted amines. In addition, a H-bond donor at the C-4 position on the isoxazole is vital for retaining the activity. Davies et al. [36] observed that S-acetamide derivatives of compounds have better bioactivity profile than the S-alkylamines. The importance of **da_amdN_6B** was further confirmed by comparing following pair of the molecules: 856 (pIC_50_ = 6.848, **da_amdN_6B** = 0) with 861 (pIC_50_ = 7.114, **da_amdN_6B** = 1). The earlier work identified the role of amide group, and in the present work, we successfully identified that a combination of amide group with H-bond donor/acceptor within six bonds is a better strategy to have better Hsp90 inhibitory activity. Therefore, such a combination of the amide nitrogen atom and H-bond donor/acceptor should be retained in future optimizations. 

In short, three molecular descriptors emphasize the importance of ring carbon atoms, especially aromatic carbon atoms. This could be attributed to the lipophilic character of the active site of Hsp90. Likewise, four molecular descriptors underline the significance of different types of nitrogen atoms, which are responsible for the establishment of the polar or H-bond interactions with polar residues and water molecules present inside the active site of Hsp90. Hence, the present work is successful in identifying reported as well as novel pharmacophoric features of Hsp90 inhibitors.

## 4. Materials and Methods

The OECD (Organization for Economic Cooperation and Development) guidelines and a standard protocol recommended by different researchers [11,12,13,16,18,25,26,29,30,37] involve the sequential execution of (1) data collection and its curation, (2) structure generation and calculation of molecular descriptors, (3) objective feature selection (OFS), (4) splitting the dataset into training and external validation sets, (5) subjective feature selection involving building a regression model and validation of the developed model, which have all been followed to build a widely applicable QSAR model for Hsp-90 inhibitory activity. This also ensures thorough validation and successful application of the model.

### 4.1. Data Collection and Its Curation

**Figure 6:**
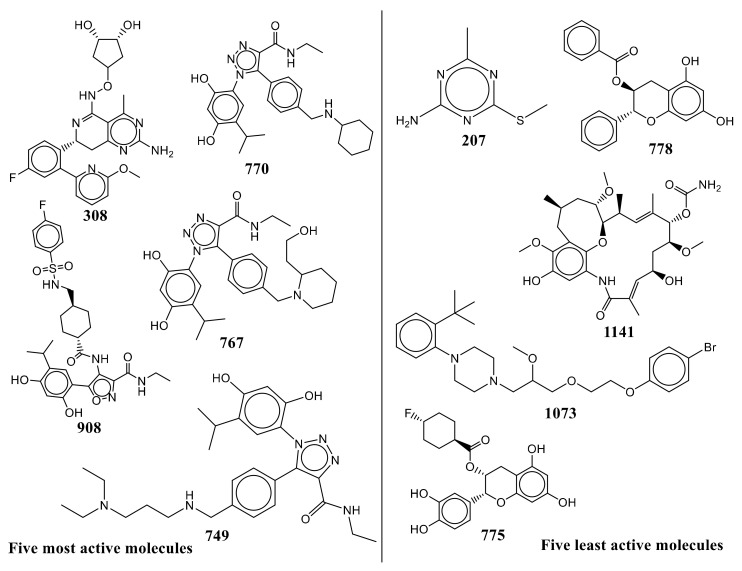
Representative examples from the selected dataset (the five most active and five least active molecules).

The dataset of Hsp-90 inhibitory activity used for building, training, and validating the QSAR model in the present work was downloaded from BindingDB (https://www.bindingdb.org/bind/index.jsp, accessed on 24 December 2021), which is a free and publicly accessible database. Initially, the dataset comprised 1839 molecules. Then, as a part of data curation, entries with ambiguous IC_50_ values, duplicates, salts, metal-based inhibitors, etc. were omitted [11,12,13,16,18,25,26,29,30,37]. The final dataset comprises 1141 structurally diverse molecules with remarkable variation in structural scaffolds, which were tested experimentally for potency in terms of IC_50_ (nM) (see the MS Excel file ‘SupplementaryMaterial-Final’ in the Supplementary Materials). The dataset includes N-terminal inhibitors of Hsp90. The experimental IC_50_ values have a sufficient variation ranging from 5 to 350,000 nM. After that, IC_50_ values were converted to their negative logarithmic value (pIC_50_ = −log_10_IC_50_) so that a comparison of their values became easier. In Table 1 and Figure 6, some of the most and least active molecules are included as examples only.

### 4.2. Calculation of Molecular Descriptors and Objective Feature Selection (OFS)

**Figure 7:**
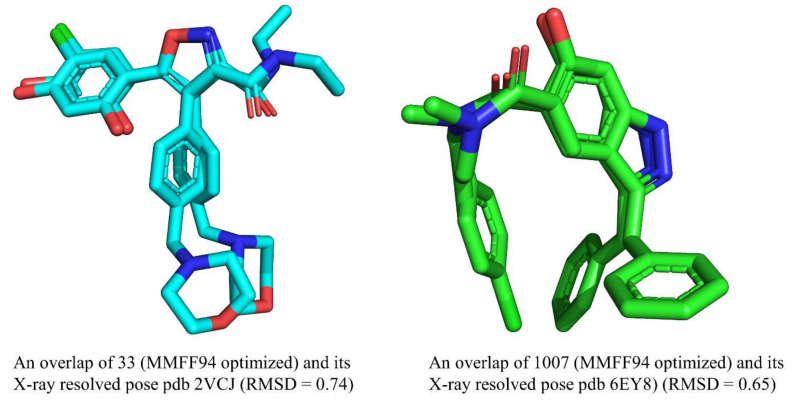
A comparison of X-ray-resolved and MMFF94-optimized structures of molecules 1007 and 33.

A crucial step before the calculation of molecular descriptors is to convert the SMILES notations to 3D-optimized structures and partial charge assignment, which was accomplished using OpenBabel 3.1 [38] using MMFF94 force field. In the present work, the X-ray-resolved structure of molecule **1007** (pdb 6YE8) was used to identify the parameter tuning in OpenBabel, required to get a better optimized structure, until there was a high similarity between the MMFF94-optimized structure and X-ray-resolved structure. This enhances the chances of getting a bioactive conformer, which in turn is highly beneficial for further optimization of Hsp90 inhibitors in the drug discovery pipeline. A comparison of the X-ray-resolved structures of molecules **1007** and **33** (pdb 2VCJ) and their respective MMFF94-optimized structures are represented in Figure 7. 

From Figure 7, it is clear that there is a high similarity between the X-ray-resolved and MMFF94-optimized structure of molecules **1007** and **33**, which indicates that appropriate parameter tuning was achieved to optimize the rest of the molecules. That is, the same parameter tuning in OpenBabel was used to optimize the other molecules of the selected dataset. The parameters are as follows: geometry optimization, steepest descent, number of steps: 1500; cut off: 0.01.

In the next step, the 3D-optimized structures of all molecules in the dataset were used to calculate a good number of molecular descriptors. It is important to note that calculation of diverse molecular descriptors enhances the chances of a successful QSAR analysis and significantly helps in mechanistic interpretation. However, descriptor pruning is very useful as it further strengthens the diminished risk of overfitting from noisy redundant descriptors. To fulfil these objectives, more than 40,000 molecular descriptors were generated using *PyDescriptor* [39]. After that, OFS involved elimination of the near constant (90% molecules) and highly intercorrelated (|R| > 0.90) molecular descriptors. For this, QSARINS-2.2.4 was used. The final set of molecular descriptors comprises 1228 molecular descriptors, which still comprise manifold descriptors (1D- to 3D-), leading to coverage of a broad descriptor space.

### 4.3. Splitting the Dataset into Training and External Sets and SFS (Subjective Feature Selection)

**Figure 8:**
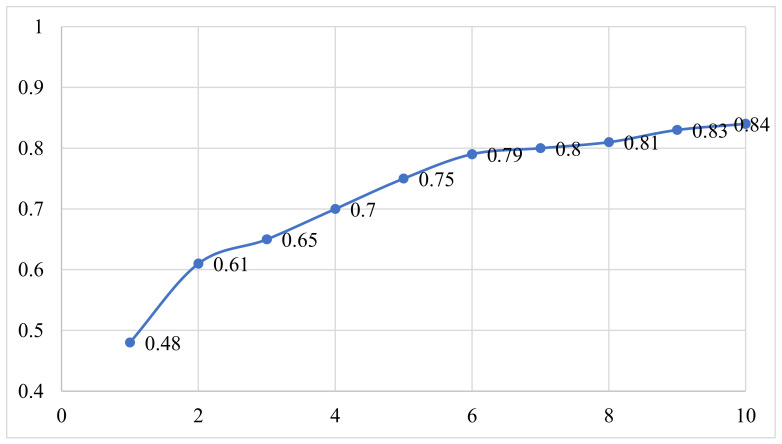
Plot of number of descriptors against leave-one-out coefficient of determination Q^2^_LOO_ to identify the optimum number of descriptors.

Subjective feature selection involves selection of appropriate number and set of molecular descriptors to build a model using suitable algorithm. Prior to SFS, it is essential to divide the dataset into training and test (also known as external or prediction set) sets with a proper composition and proportions to circumvent information leakage and to verify the predictive ability of a model [11,12,13,16,18,25,26,29,30,37]. Hence, the dataset was randomly split into training (80% = 915 molecules) and prediction or external (20% = 226 molecules) sets. It is to be noted that the training set was used for the selection of optimum number of molecular descriptors, and the sole purpose of prediction/external set was to validate the external predictive ability of the model (Predictive QSAR). A GA-MLR-based QSAR model is free from over-fitting if it comprises an optimum number of molecular descriptors. Therefore, in the present work, a simple yet effective method of identifying the breaking point was used. Generally, the continuous inclusion of molecular descriptors in the GA-MLR model significantly increases the value of Q^2^_LOO_, but after the breaking point, the value of Q^2^_LOO_ does not increase significantly [24]. The number of molecular descriptors corresponding to the breaking point was considered optimum for model building. A graph (see Figure 8) was plotted between the number of molecular descriptors involved in the model and Q^2^_LOO_ values, which indicated that the breaking point agreed with the six molecular descriptors. Consequently, QSAR models comprising more than six descriptors were not considered. For SFS, the set of molecular descriptors was selected using the genetic algorithm integrated with multilinear regression (GA-MLR) method available in QSARINS-2.2.4 (generations per size: 10,000; population size: 50; mutation rate: 60; significance level: 0.05; fitness parameter: Q^2^_LOO_).

### 4.4. Building Regression Model and Its Validation

The GA-MLR approach resulted in the generation of a good number of models having good to excellent statistical performance. Therefore, the following stringent parameters and criteria suggested by different researchers were used to select the best model [11,12,13,16,18,25,26,29,30,37,40]: R^2^_tr_ ≥ 0.6, Q^2^_loo_ ≥ 0.5, Q^2^_LMO_ ≥ 0.6, R^2^ > Q^2^, R^2^_ex_ ≥ 0.6, RMSE_tr_ < RMSE_cv_, ΔK ≥ 0.05, CCC ≥ 0.80, Q^2^-F^n^ ≥ 0.60, r^2^_m_ ≥ 0.5, (1-r^2^/r_o_^2^) < 0.1, 0.9 ≤ k ≤ 1.1 or (1-r^2^/r’_o_^2^) < 0.1, 0.9 ≤ k’ ≤ 1.1,| r_o_^2^− r’_o_^2^| < 0.3, RMSE_ex,_ MAE_ex_, R^2^_ex_, Q^2^_F1_, Q^2^_F2_, and Q^2^_F3_, and low R^2^_Yscr_, RMSE, and MAE. The details of these statistical parameters are available in the Supplementary Materials. An important aspect of validation of a QSAR model is to identify the applicability domain. In the present work, the William’s plot was plotted to assess the applicability domain of the QSAR model [11,12,13,16,18,25,26,29,30,37,41,42].

## 5. Conclusions

In the present work, a relatively large and structurally diverse dataset of 1141 Hsp90 inhibitors was used for developing a six-descriptor-based and extensively validated GA–MLR QSAR model with R^2^_tr_ = 0.78, Q^2^_LMO_ = 0.77, R^2^_ex_ = 0.78, and CCC_ex_ = 0.88. The inclusion of easily understandable descriptors resulted in identification of important pharmacophoric features that are correlated with Hsp90 inhibitory activity. The present QSAR analysis effectively captured a mixture of reported as well as novel significant structural features. The analysis vindicates that ring and aromatic carbons are important in deciding the activity. In addition, different types of nitrogen atoms in correlation with different types of carbon atoms influence the Hsp90 inhibitory activity. A good balance of external predictive ability and mechanistic interpretations, which are further supported by the reported crystal structures of Hsp90 inhibitors, make the QSAR model useful for the future optimization of molecules in the pipeline as a better Hsp90 inhibitor.

## Figures and Tables

**Table 1:** SMILES notation, IC_50_ (nM) and pIC_50_ (M) of the five most and least active molecules of the selected dataset.

S.N.	Ligand SMILES	IC_50_ (nM)	pIC_50_ (M)
308	COc1cccc(n1)-c1cc(F)ccc1[C@H]1Cc2nc(N)nc(C)c2C(NOC2C[C@H](O)[C@H](O)C2)=N1	5	8.301
908	CCNC(=O)c1noc(c1NC(=O)[C@H]1CC[C@H](CNS(=O)(=O)c2ccc(F)cc2)CC1)-c1cc(C(C)C)c(O)cc1O	5.4	8.268
770	CCNC(=O)c1nnn(c1-c1ccc(CNC2CCCCC2)cc1)-c1cc(C(C)C)c(O)cc1O	6.8	8.167
767	CCNC(=O)c1nnn(c1-c1ccc(CN2CCCCC2CCO)cc1)-c1cc(C(C)C)c(O)cc1O	10	8
749	CCNC(=O)c1nnn(c1-c1ccc(CNCCCN(CC)CC)cc1)-c1cc(C(C)C)c(O)cc1O	12	7.921
775	Oc1cc(O)c2C[C@@H](OC(=O)[C@H]3CC[C@H](F)CC3)[C@H](Oc2c1)c1ccc(O)c(O)c1	69,000	4.161
1073	COC(COCCOc1ccc(Br)cc1)CN1CCN(CC1)c1ccccc1C(C)(C)C	70,430	4.152
1141	CO[C@H]1C[C@H](C)Cc2c(OC)c(O)cc3NC(=O)\C(C)=C\[C@H](O)C[C@H](OC)[C@@H](OC(N)=O)\C(C)=C\[C@H](C)[C@H]1Oc23	96,000	4.018
778	Oc1cc(O)c2C[C@H](OC(=O)c3ccccc3)[C@H](Oc2c1)c1ccccc1	120,000	3.921
207	CSc1nc(C)nc(N)n1	350,000	3.456

## Data Availability

Data is contained within the article and Supplementary Materials.

## Supplementary Materials

The following are available online at https://www.mdpi.com/article/10.3390/ph15030303/s1.

## Abbreviations

SMILES	Simplified molecular-input line-entry system
GA	Genetic algorithm
MLR	Multiple linear regression
QSAR	Quantitative structure−activity relationship
WHO	World Health Organization
ADMET	Absorption, distribution, metabolism, excretion, and toxicity
OLS	Ordinary least square
QSARINS	QSAR Insubria
OECD	Organization for Economic Cooperation and Development
