# Supplementary material for: Exploring the Prominent and Concealed Inhibitory Features for Cytoplasmic Isoforms of Hsp90 Using QSAR Analysis
Source: Pharmaceuticals (Basel). 2022 Mar 1;15(3):303. doi: 10.3390/ph15030303 (PMC8953649; doi:10.3390/ph15030303)
Supplement: Supplementary file 1 [file pharmaceuticals-15-00303-s001.zip › Supplementary Material.pdf]

Exploring the Prominent and Concealed Inhibitory Features for Cytoplasmic Isoforms of Hsp90 Using QSAR Analysis

Magdi E. A. Zaki [1,*], Sami A. Al-Hussain [1], Syed Nasir Abbas Bukhari [2], Vijay H. Masand [3,*], Mithilesh M. Rathore [3], Sumer D. Thakur [4] and Vaishali M. Patil [5]

[1] Department of Chemistry, Faculty of Science, Imam Mohammad Ibn Saud Islamic University,
Riyadh 13318, Saudi Arabia; sahussain@imamu.edu.sa
[2] Department of Pharmaceutical Chemistry, College of Pharmacy, Jouf University,
Al jouf 72388, Saudi Arabia; sbukhari@ju.edu.sa
[3] Department of Chemistry, Vidya Bharati Mahavidyalaya, Amravati 444 602, Maharashtra, India;
mithsrathore@gmail.com
[4] Department of Chemistry, RDIK and NKD College, Badnera-Amravati 444 701, Maharashtra, India;
sdthakur11@gmail.com
[5] Department of Pharmaceutical Chemistry, KIET School of Pharmacy, KIET Group of Institutions,
Delhi-NCR, Ghaziabad 201206, Uttar Pradesh, India; vaishuwise@gmail.com
* Correspondence: mezaki@imamu.edu.sa (M.E.A.Z.); vijaymasand@gmail.com (V.H.M.)

Statistical symbols with names and explanations:

R^2 – correlation coefficient, Q^2 – leave-one-out 'crossvalidated R^2', R^2_{adj} - adjusted R^2, SEE – standard error of estimates, RMSE - root mean squared error, MAE - mean absolute error, CCC - concordance correlation coefficient, for the training (tr), and test (ex) sets; R^2_{LMO} and Q^2_{LMO} – leave many-out correlation coefficient and cross-validation coefficients; R^2_{Yrand} and Q^2_{Yrand} – Y- scramble correlation and cross-validation coefficients;

Statistical parameters for used for validation of QSAR models:

$$R^2 = 1 - \frac{\sum(y_i - \hat{y}_i)^2}{\sum(y_i - \bar{y})^2}$$

where y_i are the observed values of the response, ȳ the corresponding average, ŷ are the calculated values

$$Q^2 = 1 - \frac{\sum(y_i - \hat{y}_i)^2}{\sum(y_i - \bar{y})^2}$$

where y_i are the observed values of the response, ȳ the corresponding average, ŷ are the values predicted for each object when it is not in the training set.

$$Q^2_{F1} = 1 - \frac{\sum_{i=1}^{n_{EXT}}(y_i - \hat{y}_i)^2}{\sum_{i=1}^{n_{EXT}}(y_i - \bar{y}_{TR})^2}$$

where y_i are the observed values of the response, ȳ the corresponding average, ŷ are the calculated values

$$Q_{F2}^2 = 1 - \frac{\sum_{i=1}^{n_{EXT}}(y_i - \hat{y}_i)^2}{\sum_{i=1}^{n_{EXT}}(y_i - \bar{y}_{EXT})^2}$$

where y_i are the observed values of the response, ȳ the corresponding average, ŷ are the calculated values

$$Q_{F3}^2 = 1 - \frac{\left[\sum_{i=1}^{n_{EXT}}(y_i - \hat{y}_i)^2\right]/n_{EXT}}{\left[\sum_{i=1}^{n_{TR}}(y_i - \bar{y}_{TR})^2\right]/n_{TR}}$$

where y_i are the observed values of the response, ȳ the corresponding average, ŷ are the calculated values

$$CCC = \frac{2\sum_{i=1}^{n_{EXT}}(y_i - \bar{y})(\hat{y}_i - \bar{\hat{y}})}{\sum_{i=1}^{n_{EXT}}(y_i - \bar{y})^2 + \sum_{i=1}^{n_{EXT}}(\hat{y}_i - \bar{\hat{y}})^2 + n_{EXT}(\bar{y} - \bar{\hat{y}})^2}$$

$$k = \frac{\sum_{i=i}^{n_{EXT}} y_i \hat{y}_i}{\sum_{i=1}^{n_{EXT}} \hat{y}_i^2}$$

$$k' = \frac{\sum_{i=i}^{n_{EXT}} y_i \hat{y}_i}{\sum_{i=1}^{n_{EXT}} y_i^2}$$

$$r_m^2 = r^2\left(1 - \sqrt{r^2 - r_0^2}\right)$$

$$\overline{r_m^2} = \frac{(r_m^2 + r_m'^2)}{2}$$

$$RMSE = \sqrt{\frac{\sum_{i=1}^{n_{EXT}}(y_i - \hat{y}_i)^2}{n_{EXT}}}$$

$$MAE = \frac{\sum_{i=1}^{n_{EXT}}|y_i - \hat{y}_i|}{n_{EXT}}$$

where y_i are the observed values of the response, ȳ the corresponding average, ŷ are the calculated values
